# Comparison of apical and coronal extrusions using reciprocating and rotary instrumentation systems

**DOI:** 10.1186/s12903-015-0081-z

**Published:** 2015-08-07

**Authors:** Yan Lu, Min Chen, Feng Qiao, Ligeng Wu

**Affiliations:** Department of Endodontics and restorative dentistry, School of Stomatology, Tianjin Medical University, Tianjin, 300070 China; Department of Maxillofacial Surgery, School of Stomatology, Tianjin Medical University, Tianjin, 300070 China

## Abstract

**Background:**

The aim of this study was to compare the apical and coronal extrusions by using two reciprocating and two rotary instrumentation systems.

**Methods:**

Eighty extracted human single-rooted anterior teeth were randomly assigned to four groups. Four different root canal instrumentation systems were used according to the manufacturers instructions, including two reciprocating single-file systems, Reciproc and WaveOne, and two full-sequence rotary BLX and ProTaper instruments. Debris collected from the coronal by the instruments and apical extrusions were quantified respectively. After drying the collections, the mean weight of debris collected from apical and coronal extrusions was assessed using an electronic balance and analyzed using the Kruskal–Wallis H and Mann–Whitney *U* tests.

**Results:**

Statistically significant differences in the apical extrusion were observed among the four groups. Reciproc and WaveOne instruments produced significantly less debris than BLX and ProTaper instruments (*P <* 0.05).

**Conclusions:**

All of the systems produced apical extrusion of debris. However, reciprocating single-file systems produced less apical extrusion than full-sequence rotary systems. No relationship was observed between apical and coronal extrusions.

## Background

Root canal therapy is the most effective method of treating pulpitis and periapical periodontitis. The main purpose of root canal instrumentation is to enlarge the root canal system to remove the residual pulp tissue and bacteria as well as to provide a space for the delivery of root canal irrigants, medicaments, and finally root-filling materials. During root canal preparation, dentin filings, pulp tissue fragments, necrotic tissue, microorganisms, and irrigants may be extruded into the periradicular tissues despite strict control of the working length [1, 2]. The resulting apical extrusions have potential to disrupt the balance between microbial aggression and host defenses, leading to episodes of postoperative complications [3]. The so called “flare-up” is described as the occurrence of pain, swelling, or a combination of both during root canal treatment [3, 4]. The incidence of flare-up is reported to range between 1.4 % and 16 % [5].

All preparation techniques and instrumentations have been reported to be associated with apical extrusions, even when the apical terminus is avoided [6–12]. In 1975, Vande Visse and Brilliant [13] firstly quantified the amount of apical extruded debris during instrumentation. They concluded that instrumentation with an irrigant produced extrusions while without an irrigant produced no collectible debris. Currently, a common finding is that the push-pull filing motion tend to produce more apical extrusions than instrumentation techniques with a rotational force [7, 10, 11]. This may be because rotary instruments have a tendency to pull debris into the flutes, thus leading them out of the root canal in a coronal direction [14].

The most significant feature of the ProTaper system is the taper gradual increase from the tip of the instrument up to the shaft. The convex triangular cross-section is another unique characteristic of ProTaper, which reduces the area of contact between the file and the dentinal walls. This system consists of three shaping files (SX, S1, S2) and three finishing files (F1, F2, and F3).

BLX (B&L Biotech, Seoul, Korea) is the newest product from the B&L Biotech company. This system is designed to achieve “biological sizes” in an efficient and safe manner which consists of three files (15/.06, 25/.06, 35/.04). BLX instruments possess alternating cutting edges, non-cutting safety tips, sharp cutting edges without radial lands, and electro-chemical surface treatment.

Recently, two new single-file nickel-titanium (NiTi) systems, Reciproc (VDW, Munich, Germany) and WaveOne (Dentsply Maillefer, Ballaigues, Switzerland), have come forward the market. These systems claim to be able to complete root canal preparation with only one instrument. The files are made of a special NiTi alloy known as M-Wire, which is created by an innovative thermal treatment process. This improvement increases the flexibility and resistance to cyclic fatigue [15–17]. A recent study has shown that in canals with a high prevalence of isthmuses and protrusions, multifile rotary systems may be preferred over reciprocating files because they can yield cleaner canals with less accumulation of debris [18]. Bürklein and Schäfer [19] evaluated apically extruded debris using the reciprocating single-file systems WaveOne and Reciproc and the Mtwo and ProTaper full-sequence rotary instrumentation systems. They found that the reciprocating files produced significantly more debris than the rotary systems with Reciproc producing the greatest amount of debris.

Most studies of reciprocating single-file systems have evaluated their mechanical characteristics. However, few studies have focused on their potential for producing apical extrusions. Moreover, there has been no published study of the BLX system and the coronal extrusion collected by various insruments to date. This study compared the amount apical extrusion and coronal one from the dentine wall when two reciprocating single-file systems, Reciproc and WaveOne, and two rotary full-sequence systems, BLX and ProTaper, were used for root canal preparation. The study aimed to compare the apical and coronal extrusions by using two reciprocating and two rotary instrumentation systems. The null hypothesis was that there was no relationship between the apical and coronal extrusion of debris.

## Methods

A total of 80 freshly extracted human anterior teeth with mature apices and straight root canals (<10°) were selected. This study was approved by the Medical Ethics Committee of Tianjin Medical University and informed consent was obtained from all patients. The informed consent was written and the consent procedure was approved by the ethics committee, the ethics statement that our research has been conducted in full accordance with the World Medical Association Declaration of Helsinki. Only single-rooted teeth with a single canal and a single apical foramen were included. These characteristics were verified by Cone Bean Computed Tomography. The teeth were subsequently stored in 0.1 % thymol solution after removing the calculus and periodontal ligaments. The total length of each tooth was measured with a Vernier caliper. Coronal access was achieved by using diamond burs, and apical patency was controlled with size 15 K-file (Dentsply Maillefer, Ballaigues, Switzerland). The width of the root canal near the apex was generally compatible with a size 15 file. The working length (WL) was obtained by measuring the length of the initial instrument (size 15) at the apical foramen minus 1 mm. A hundred and sixty microcentrifuge tubes with the volume of 1.5-mL were collected, with two tubes from each group. All of the tubes were numbered and weighed using an electronic balance with an accuracy of 0.00001 g.

### Establishment of the experimental model

**Fig. 1:**
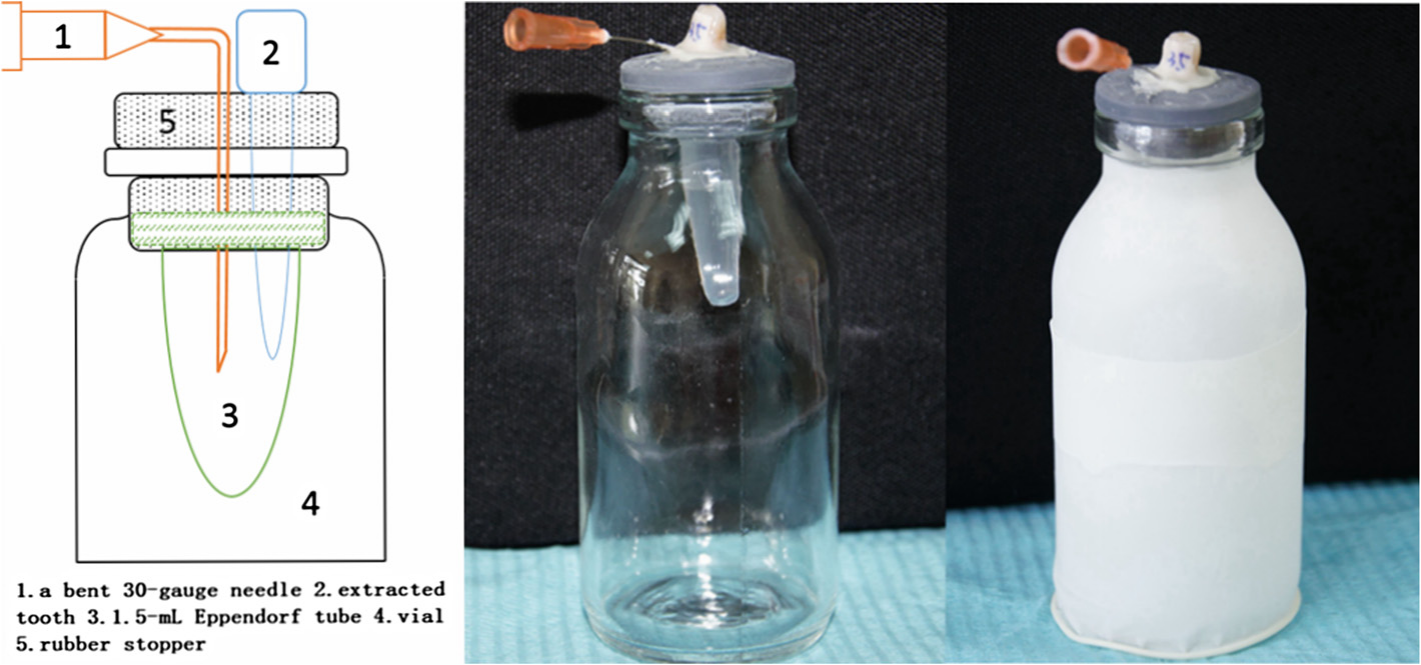
The schematic and experimental model system used to evaluate apical and coronal extrusions

Vials with rubber stoppers were adjusted by using a sharp instrument to create a hole approximately 0.3-mm in diameter through the center of each stopper [7, 20–22]. As shown in Figure 1, each tooth was inserted under pressure into the rubber stopper, which was then fixed to the cementoenamel junction by means of glass ionomer cement. The 1.5-mL microcentrifuge tubes were then inserted under pressure through the stoppers. A bent 30-gauge needle was forced alongside the stopper for use as a drainage cannula to balance the air pressure inside and outside the microcentrifuge tubes. The vials were shielded from the operator by a rubber-dam during the instrumentation process.

### Canal instrumentation and apical extrusion collection

The 80 extracted teeth were randomly assigned to four groups of 20 teeth respectively, with 10 maxillary and 10 mandibular anterior teeth in each group. The root canals were instrumented with the Reciproc, WaveOne, BLX, and ProTaper systems in accordance with the manufacturers’ recommendations. No glide path was created before instrumentation as the initial size of all canals equal to size 15.

#### Reciproc group

A R25 Reciproc file with a size 25 tip and a taper of 0.08 over the first 3 mm was used in a reciprocating slow in-and-out pecking motion(full working length). The flutes of the instrument were cleaned after three pecks.

#### Waveone group

A primary WaveOne file with a size 25 tip and a taper of 0.08 was used in a reciprocating slow in-and-out pecking motion(full working length). The flutes of the instrument were cleaned after three pecks.

#### BLX group

All BLX instruments were used to the full WLs using a gentle in-and-out motion. The instrumentation sequence was #15/.06, #25/.06, and #35/.04(full working length). The motor was set at 550 rpm for speed, and the torque was 1.5 Ncm.

#### ProTaper group

ProTaper instruments were used with a gentle in-and-out motion. The sequence was as follows: SX (two thirds of the workin length), S1, S2, F1, and F2 (full working length).

The instrumentation sequence was S1 to negotiate the canal without reaching the WL; SX at two-thirds of the WL; S1 and S2 at the WL; and then F1, F2, and F3 at the WL. Once the instrument had been negotiated to the end of the canal and rotated freely, it was removed.

During the instrumentation procedure, after three pecks with the reciprocating files or after each instrument was used for the rotary systems, 1 mL of normal saline (NS) was used as an irrigant for 1 min. The irrigation needle (NaviTip 31ga; Ultradent, South Jordan, UT) was placed as deep as possible inside the canal without encountering resistance and was not deeper than the predetermined WL minus 1 mm. Meanwhile, the files were taken out of the root canal carefully, then immersed in other 1.5-mL microcentrifuge tubes with 1 mL NS that had the same number as the debris collectors. An EndoActivator with a maximum speed of 10,000 cpm was used to vibrate the tubes for 30 s. After instrumentation, the canals were irrigated with 5 mL NS for 1 min. Each tooth was then separated from the microcentrifuge tubes and the debris adhering to the apical root surface was collected by washing the apex with 0.1 mL of NS in the microcentrifuge tubes. The containing the apical extrusion and coronal extrusion were then stored in a vacuum centrifugal drying apparatus at 70 °C for 6 h at 1500 rpm. An electronic balance with an accuracy of 0.00001 g was used to weigh the tubes containing the dry debris. Three consecutive weights with a difference of <0.00002 g were obtained for each tube, and the mean value was calculated. The dry weights of the apical and coronal extrusion were then calculated by subtracting the weights of the empty tubes from the weights of the tubes containing the debris.

### Statistical analysis

The data were analyzed using SPSS 17.0 software. The Kruskal–Wallis H test was used for the comparison of groups, and multiple comparisons of groups were performed using the Mann–Whitney *U* test with the Bonferroni correction. The level of statistical significance was set at *P* = 0.05.

## Results

There was no statistically significant relationship between debris collected from apical and coronal extrusion (*P* > 0.05) when the four different types of root canal instrumentation systems were used (Table 1).

**Table 1:** Amount of Apical and Coronal Extruded Debris of Tested Instruments

	Instrumentation	M	SD	Test Statistic	*P* value
coronal extrusion debris	Reciproc	0.01129	0.00286	6.461	0.091
	WaveOne	0.01053	0.00229		
	BLX	0.01078	0.00192		
	ProTaper	0.01155	0.00581		
apical extrusion debris	Reciproc	0.00505	0.00152	22.039	0.000*
	WaveOne	0.00536	0.00145		
	BLX	0.00840	0.01185		
	ProTaper	0.00981	0.00216		

M: mean

SD: standard deviation

*Statistically significant difference *p* < 0.05

**Fig. 2:**
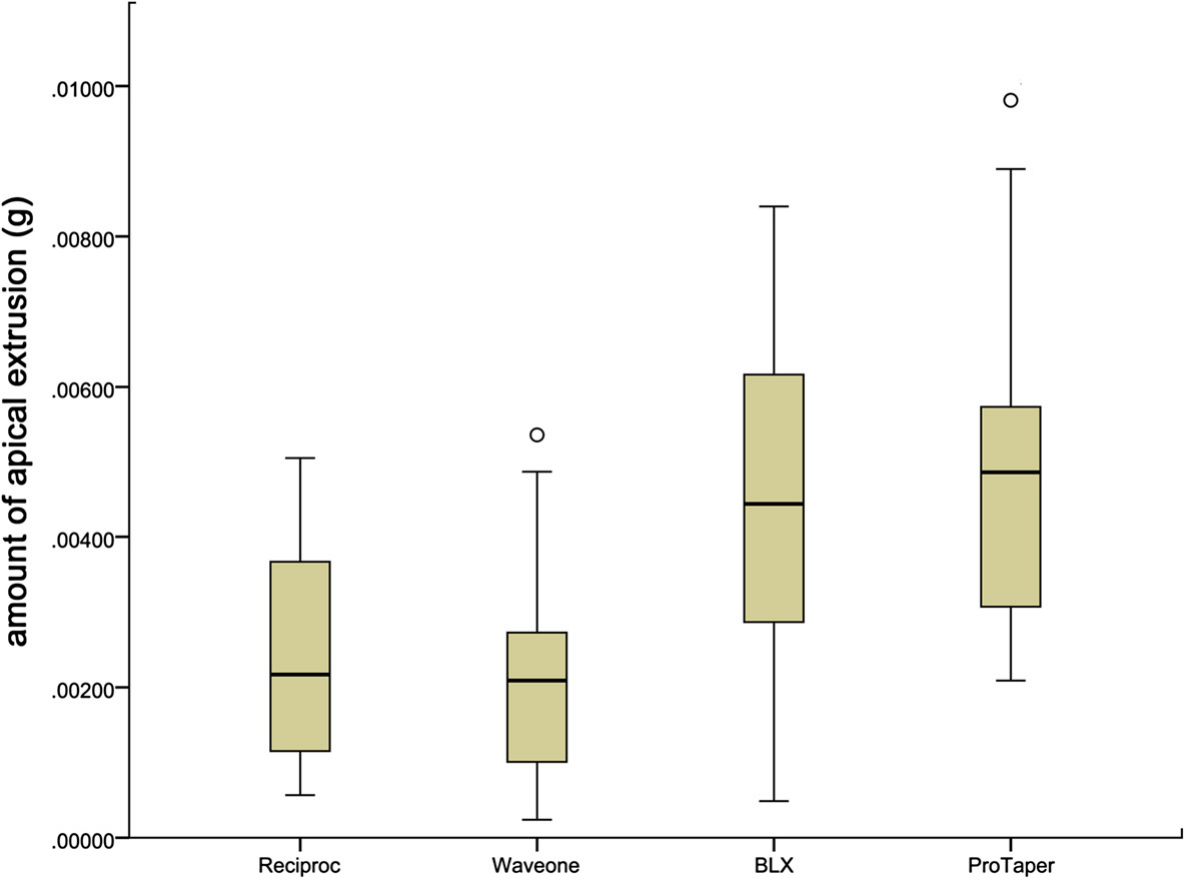
Box plots of amount of apical extrusion of the four instrumentation systems, illustrating the median, minimum, and maximum values, as well as the standard deviation data of each experimental group

**Fig. 3:**
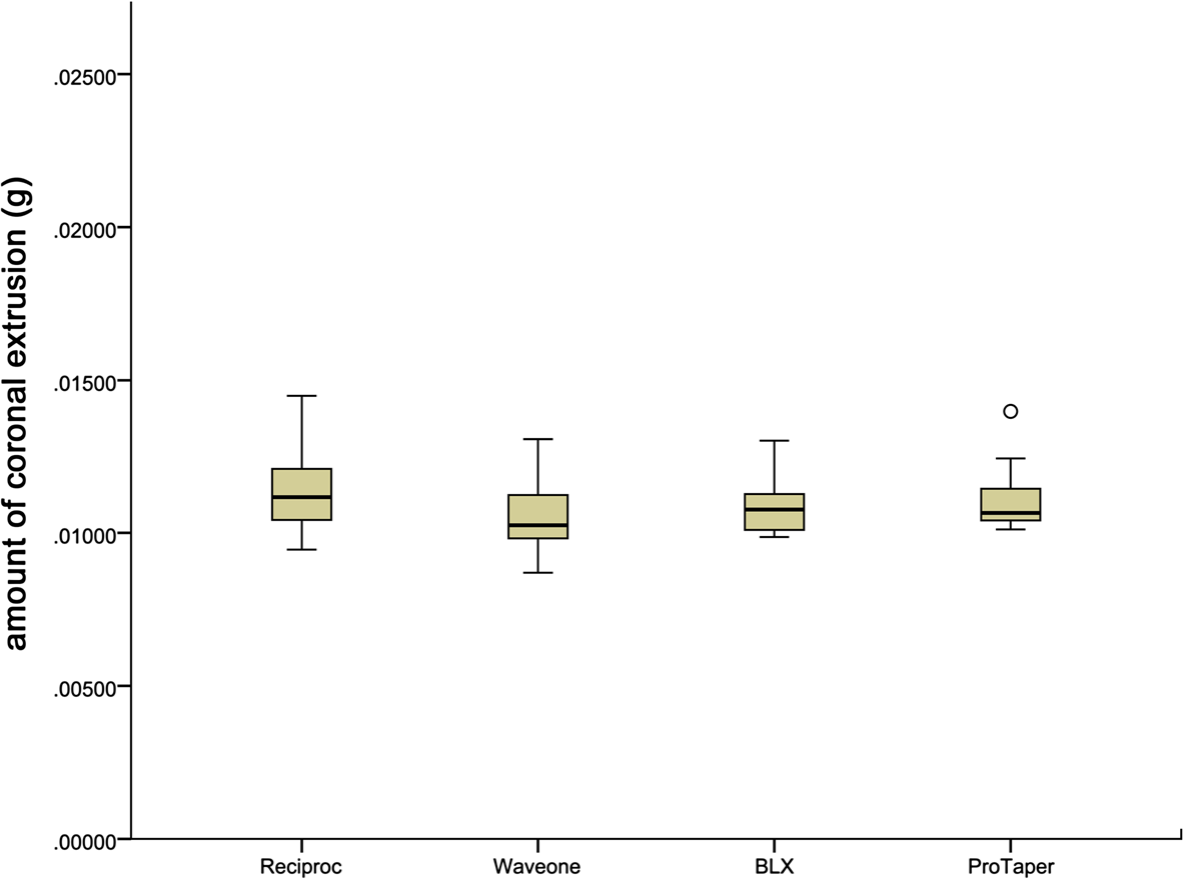
Box plots of amount of coronal extrusion of the four instrumentation systems, illustrating the median, minimum, and maximum values, as well as the standard deviation data of each experimental group

However, as shown in Fig. 2, statistically significant differences in apical extrusion of debris and irrigants were observed among the four systems. The rotary BLX and ProTaper instruments were associated with greater amounts of apical extrusion debris than the Reciproc and WaveOne systems (*P* < 0.05). No significant differences were observed between the two reciprocating systems, Reciproc and WaveOne, or between the two rotary systems, BLX and ProTaper (*P* > 0.05). Moreover, no significant differences in apical extrusion between maxillary and mandibular teeth was observed among the four groups (*P* > 0.05).As shown in Fig. 3, there was no significant difference in coronal extrusion (*P* > 0.05) among the four instruments. However, significant differences in coronal extrusion between maxillary and mandibular teeth were observed among the three groups (*P* < 0.05), except for with the Reciproc system (*P* > 0.05) (Table 2).

**Table 2:** Amount of Apical and Coronal Extruded Debris of Maxillary and Mandibular Teeth

		M	SD	Test Statistic	*P* value
coronal extrusion debris	maxillary	0.01059	0.00167	10.045	0.002*
	mandibular	0.01145	0.00230		
apical extrusion debris	maxillary	0.00488	0.00825	0.193	0.660
	mandibular	0.00343	0.00233		

M : mean

SD: standard deviation

*Statistically significant difference *p* < 0.05

**Table 3:** Relationship between apical and coronal extruded debris of the total tooth length or WL

	Total length		WL	
	Correlation	P value	Correlation	*P* value
coronal extrusion debris	−0.451	0.000*	−0.480	0.000*
apical extrusion debris	0.104	0.358	0.004	0.97

*Statistically significant difference *p* < 0.05

No significant relationship was observed between apical extrusion of debris and the total tooth length or WL (*P* > 0.05). Coronal extrusion was negatively correlated with both lengths (*P* < 0.05) (Table 3).

## Discussion

During the root canal preparation procedures, infected debris can be extruded into periradicular tissues, which may be one of the most important causes of postoperative pain. There are two types of factors that can affect such extrusions: firstly, natural physical factors, such as the anatomy of the apical constriction [2, 8] dentin hardness [10], and quantity and momentum of flow of the irrigant [7], and secondly, mechanical factors, such as the selection of the final apical size of the instrument [23], and instrumentation techniques [24]. In addition, the size of the irrigation needle and its depth into the canal may affect the quantity of extrusion [25]. This investigation aimed to compare the amount of apical and coronal extrusion of debris after preparation of straight root canals using the new reciprocating single-file Reciproc and WaveOne systems and the rotary full-sequence BLX and ProTaper instruments.

Our findings shown that the single-file systems Reciproc and WaveOne, extruded less debris than the full-sequence BLX and ProTaper, a finding that differed from that reported by Bürklein and Schäfer [19]. This may be related to the apical size of the extracted teeth after instrumentation. Bürklein and Schäfer instrumented the teeth to size 40. As Al-Omari and Dummer [6], McKendry [10], and Fairbourn et al. [26] reported that no significant correlation was found between apical size and the amount of debris extruded, this study did not control apical size after instrumentation, but prepared the root canal according to the manufacturers’ recommendations.

Previous research has focused on the quantity of apically extruded debris, while no study has assessed the amount of debris cut from the dentin wall of the root canal and carried out of the orifice. This study used an auxiliary irrigation device EndoActivator to release the debris by acoustic shocking. Dentine debris that adhered to the instrument flutes was transferred into an microcentrifuge tubes, and this was then used to assess the cutting and debris-collecting abilities of the different instruments. The reciprocating single-file instruments resulted in less apical extrusion than the rotary full-sequence systems. However, there was no difference in coronal extrusion among the four preparation systems, and no relationship between apical and coronal extrusions was observed. These findings may be related to the movement, cross-sectional design, and thread pitch of the instruments. Reciproc is designed similar to the traditional Mtwo, which has an S-shaped cross-section design with a larger space to accommodate dentine debris [27]. WaveOne, BLX, and ProTaper use a triangular or improved cross-section design and have a relatively less available space. The tip of the WaveOne has a debris diversion trench on each cutting edge, which reduces the tendency for debris extrusion into periradicular tissues. Because of the reciprocating motion, Reciproc and WaveOne are better at squeezing debris into the flutes and carrying the debris out of the root canal orifice, thereby reducing apical extrusion of debris. In contrast, the rotary BLX instruments are more likely to exert a spiral effect which may push the debris out of the apical foramen. This study also found that apical extrusion was not associated with the total length of the tooth or the WL, which is consistent with the report by Fairbourn et al. [26]. However, coronal extrusion was negatively correlated with both lengths. The reason might be that when the instrument was removed from the root canal, some debris may fell off the thread and stuck to the root canal wall. Therefore, the longer the root canal, the lesser the debris is removed from the orifice. Although the average thread pitches of Reciproc and WaveOne were greater than those of BLX and ProTaper, resulting in more debris being squeezed into the flutes, the contact area between the debris and root canal wall was larger and more debris was stuck on the root canal wall. As a result, there was no difference in coronal extrusion among the four systems. Therefore, abundant chemical irrigation must be combined with mechanical instrumentation to remove the debris more thoroughly during root canal preparation.

The research objects selected for this study were 80 fresh extracted teeth, 40 from each jaw, and each system was used to instrument 10 maxillary and 10 mandibular anterior teeth. Apical extrusion was not found to be related to tooth position in our study. The average diameters of the root canals of maxillary anterior teeth are greater than those of mandibular teeth, therefore it can be speculated that apical extrusion of debris maybe not related to the diameter of the root canal.

In this experiment, physiological saline was used as the root canal irrigant but not the internationally recognized combination of NaOCl/EDTA. Studies have shown that NaOCl and EDTA interact with each other and produce a chemical reaction to generate chelate [28]. This is also why saline is used to flush the root canal between the applications of the two chemicals. In the preliminary experiments for this study, 3 % NaOCl was used after each instrument and 17 % EDTA was used as the final irrigant. Multiple flocculent precipitates were then found in the microcentrifuge tubes that contained the apical extrusion, which affected the accuracy of the experimental findings. Therefore, in the present study the 0.9 % NaCl solution was used as the root canal irrigant. Some scholars might consider the salt precipitation that occurs after desiccation of the NaCl solution, a factor that interferes with the experimental findings [29]. Under the actual condition, apical extrusion include debris as well as irrigants and there would not be any extrusions without irrigation of the root canal [13]. Therefore, the 0.9 % NaCl solution was used in this experiment and after drying, the tubular contents included apically extruded debris incorporate with NaCl salt precipite. This precipitate can be used as an indirect measure of the extruded irrigants and thus increases the accuracy of the apical extrusion measurements.

Further studies are required to determine whether these *in vitro* experimental results can be applied to clinical practice. Biological periapical tissue can exert a certain amount of pressure *in vivo* and is able to resist debris and irrigant extrusion. However, in the established experimental model, the microcentrifuge tubes used to collect the extrusion was linked to the outside barometric pressure, and the effect of gravity would have prompted the irrigants to move out of the apical foramen along with the dentin debris. This is a shortcoming of *in vitro* designs because they have no periapical resistance, as was already discussed by Myers and Montgomery [30]. Recently, some researchers have used a floral foam to simulate the periapical tissue environment [25]. However, the foam can absorb debris and irrigants, thereby possibly affecting the accuracy of the experimental findings. Psimma et al. [31] used a new method to accurately measure apically extruded irrigants, but they were unable to assess extruded debris at the same time and this complicates model building. Therefore, we did not attempt to simulate the periapical tissue environment in this study.

## Conclusions

1. Under the conditions of this study, all instrumentation systems caused apical extrusion of debris and irrigants.
2. The use of reciprocating single-file systems resulted in less apical extrusion of debris than full-sequence rotary instruments.
3. The null hypothesis that apical extrusion of debris by root canal preparation systems is not associated with coronal extrusion was confirmed.
